# The Mentalisation Switch: Therapist Reflective Capacity and Alliance Dynamics in Digital MCT+ for Bipolar Disorder—A Longitudinal Quantitative Case Series

**DOI:** 10.1002/cpp.70260

**Published:** 2026-03-19

**Authors:** Roberto Maluenda‐Gatica, Christian Araya, Javier Morán‐Kneer, Ulises Ríos, Steffen Moritz, Angus MacBeth

**Affiliations:** ^1^ Department of Clinical Psychology, School of Health in Social Science University of Edinburgh Edinburgh UK; ^2^ Institute of Statistics, Faculty of Science Pontificia Universidad Católica de Valparaíso Valparaíso Chile; ^3^ School of Psychology Universidad de Valparaíso Valparaíso Chile; ^4^ Department of Psychiatry, School of Medicine University of Valparaíso Valparaíso Chile; ^5^ Department of Psychiatry and Psychotherapy University Medical Center Hamburg‐Eppendorf Hamburg Germany

**Keywords:** bipolar disorder, digital psychotherapy, MCT+, mentalisation, metacognitive therapy, reflective functioning, therapeutic alliance, therapist mentalisation

## Abstract

**Objective:**

This study conducted a preliminary naturalistic effectiveness evaluation of Individualised Metacognitive Therapy (MCT+) delivered via videoconferencing for individuals with bipolar I disorder (BD‐I) in a real‐world clinical setting in Chile. It also explored how therapist characteristics—specifically mentalisation capacity—influence the therapeutic alliance in digital psychotherapy.

**Methods:**

A longitudinal quantitative case series design was implemented across 14 therapist–patient dyads. Patients received 12 weekly sessions of MCT+ online. Standardised measures assessed anxiety (GAD‐7), depression (PHQ‐9), metacognitive beliefs (MCQ‐30), psychological distress (CORE‐10) and quality of life (WHOQOL‐BREF). Therapeutic alliance was tracked session‐by‐session (WAI‐S). Therapist mentalisation and attachment were evaluated at baseline (MASC‐SP, RFQ‐8, ECR‐12). Changes in outcome measures were analysed using paired *t*‐tests, effect sizes (Cohen's *d*), correlations, reliable change indices (RCI) and hierarchical linear modelling (HLM).

**Results:**

Across the intervention, there were significant decreases in anxiety (*d* = 0.64) and improvements in metacognitive beliefs (*d* = 0.37). Depression showed a modest improvement (*d* = 0.34), while changes in quality of life were negligible (*d* = −0.21). Hierarchical modelling indicated a significant interaction between automatic and controlled mentalisation (*b* = −0.45, *p* = 0.008), suggesting that flexible adjustment supported therapeutic alliance development. Attachment style showed no significant associations with the alliance (largest unadjusted effect: *ρ* = −0.54, *p* = 0.073; all adjusted *ps* > 0.99).

**Conclusions:**

Digital MCT+ showed preliminary effectiveness in reducing anxiety and maladaptive metacognitive beliefs among individuals with BD‐I, with more limited effects on depression and quality of life. Importantly, therapist mentalisation flexibility—the capacity to shift between automatic and controlled modes, or the *mentalisation switch*—emerged as a central mechanism for alliance building and engagement in digital contexts, highlighting a key target for clinical training and future research.

## Summary

- Digital MCT+ may be a useful adjunctive intervention for individuals with BD‐I, particularly for reducing anxiety and maladaptive metacognitive beliefs.
- Therapeutic alliance in videoconferencing psychotherapy appears to be shaped not only by technique, but by therapists' capacity to flexibly adjust their mentalising stance.
- Early alliance building may benefit from intuitive, affectively attuned therapist responses, whereas later phases may require more explicit, reflective mentalisation to support task and goal alignment.
- Overreliance on both intuitive and highly self‐reflective mentalising simultaneously may hinder relational attunement in digital therapy.
- Training and supervision in digital psychotherapy should attend not only to therapists' reflective capacity but also to the timing and flexibility with which different mentalising modes are deployed.

## 1 Introduction

Bipolar disorder (BD) remains a major psychiatric condition characterised by functional impairment, high comorbidity and elevated risk of suicide (Arnone et al. 2024; Berk et al. 2025; Cui et al. 2025; Glaus et al. 2025; Hu et al. 2023; Léda‐Rêgo et al. 2023, 2024; Miola et al. 2023). Importantly, beyond acute episodes, BD exerts a pervasive impact on emotional regulation, interpersonal functioning and quality of life, including during euthymic phases (Glaus et al. 2025; IsHak et al. 2024). These ongoing challenges underline the need for interventions that address not only symptomatic change but also the wider psychosocial burden of the disorder (Berk et al. 2025; Orhan et al. 2025).

Metacognitive processes have increasingly been proposed as a key mechanism contributing to persistent distress and functional impairment in BD. Metacognitive difficulties in BD include maladaptive beliefs about thinking (e.g., concerns about uncontrollability or danger of thoughts), compromised monitoring of internal states and impaired reflection on mental experiences (Favaretto et al. 2020; Østefjells et al. 2017; Popolo et al. 2017; Reinholdt‐Dunne et al. 2021; Torres et al. 2021; Van Camp et al. 2019). Such patterns have been linked to greater anxiety and depressive symptoms, low self‐compassion and distorted cognitive self‐appraisal (Palmer‐Cooper et al. 2023; Roux et al. 2021) and may remain evident even during symptom remission. This suggests that metacognition may represent a clinically meaningful and modifiable treatment target in BD.

Metacognitive training (MCT) and its individualised adaptation (MCT+) offer a structured approach designed to modify maladaptive cognitive and metacognitive processes (Moritz et al. 2023). Originally developed for psychosis, MCT focuses on identifying and correcting reasoning biases and unhelpful thinking patterns (Hotte‐Meunier et al. 2024). Meta‐analytic evidence supports its effectiveness in reducing positive symptoms and delusions in schizophrenia, with smaller effects across other symptom domains (Meinhart et al. 2025). Emerging studies also suggest that BD‐adapted formats (MCT‐BD) may improve aspects of social cognition and psychosocial functioning (de Siqueira Rotenberg et al. 2025; Haffner et al. 2018). However, empirical research on metacognitive interventions in BD remains comparatively limited, particularly in routine‐care settings and when delivered via digital modalities (Andersson et al. 2025).

In digital psychotherapy, intervention outcomes are shaped not only by therapeutic content but also by relational processes, with the therapeutic alliance (TA) being central. The TA is a robust predictor of psychotherapy outcomes across approaches and diagnoses (Flückiger et al. 2018; Horvath et al. 2011). Yet alliance formation may be especially challenging in synchronous telepsychological interventions, where reduced access to non‐verbal cues, shared physical space and embodied coregulation can constrain interpersonal attunement (Downing et al. 2021). These constraints are particularly relevant in BD, given the interpersonal sensitivities and affective dysregulation that may persist even outside acute episodes (Henry et al. 2008). Accordingly, identifying therapist factors that support alliance development in videoconferencing psychotherapy is of both theoretical and clinical importance (Andrade‐González et al. 2020).

Therapist mentalisation may represent a key mechanism supporting alliance in remote psychological treatments. Mentalisation refers to the capacity to interpret behaviour in terms of intentional mental states (Fonagy and Allison 2014; Luyten et al. 2020) and is closely linked to emotion regulation and interpersonal functioning (Choi‐Kain and Gunderson 2008; Freeman 2016). While much research has focused on patient mentalisation, therapists' capacity to mentalise—monitoring their own internal states while making sense of patients' experiences—may be particularly important for sustaining an effective therapeutic stance, especially when alliance strains emerge (Fonagy and Allison 2014; Luyten et al. 2024). From an alliance rupture–repair perspective, mentalising therapists may be better able to detect subtle shifts in engagement, regulate defensiveness and adopt a curious and validating stance that supports collaborative repair, which is linked to stronger subsequent alliance and improved outcomes (Fisher et al. 2021). Contemporary models distinguish between automatic (implicit) and controlled (explicit) mentalising modes, with clinical effectiveness potentially depending on the ability to flexibly shift between these systems under relational or cognitive strain (Ferrero et al. 2024; Lüdemann et al. 2021). This flexible deployment can be conceptualised as a *mentalisation switch*, which may be particularly relevant in videoconferencing psychotherapy where reduced interpersonal cues increase the likelihood of misattunement and place greater demands on timely rupture recognition and repair. Accordingly, digital delivery may amplify the need for rapid recalibration between implicit and explicit mentalising in order to sustain alliance continuity when interpersonal signals are attenuated.

Despite its conceptual relevance, few studies have examined whether therapist mentalising profiles—particularly the *interaction* between automatic and reflective capacities—predict alliance development over time, and this gap appears even more pronounced in digital psychotherapy contexts (Luyten et al. 2024). To address these limitations, the present study evaluated MCT+ (Balzan et al. 2019) delivered via videoconferencing to euthymic individuals with BD‐I in Chile using a longitudinal quantitative case series design. We hypothesised that (H1) patients would show improvements in anxiety and metacognitive beliefs following digital MCT+; (H2) therapists' MASC and RFQ scores are hypothesised to predict alliance levels and their evolution across sessions, reflecting meaningful differences between therapist–patient dyads; and (H3) a significant MASC × RFQ interaction would predict alliance development over time, such that mentalising flexibility (rather than high levels of both capacities) would be associated with stronger alliance trajectories.

## 2 Method

### 2.1 Design

A longitudinal case series design with repeated measures was used to explore within‐subject change trajectories in outpatient settings. We recruited therapist–patient pairs from May 2021 to September 2022.

### 2.2 Participants

Participants were referred through a specialised tertiary bipolar disorder programme within a public psychiatric hospital in Chile. Although four patients were not receiving treatment directly within the hospital, they remained under the regular outpatient care of the programme's lead psychiatrist, who was responsible for their clinical management.

Inclusion criteria for patients were (1) aged 18–65 years; (2) DSM‐5 diagnosis of bipolar I disorder confirmed by a licensed psychiatrist and the Young Mania Rating Scale‐SP score ≤ 12 (Colom et al. 2002); (3) fluency in Spanish; and (4) access and capacity to engage in videoconferencing therapy. Exclusion criteria were (1) severe learning disabilities or pervasive developmental disorders; (2) significant language difficulties; and (3) concurrent participation in other psychological treatments.

Participants ranged in age from 19 to 57 years (*M* = 33.1, SD = 10.1), and all were Chilean nationals (see Table 1). Most resided in urban areas, specifically Valparaíso (50.0%) and Viña del Mar (21.4%), with the remainder residing in rural settings. The average duration since diagnosis of bipolar I disorder was 11.9 years (SD = 7.9), with age of onset ranging from 5 to 36 years (*M* = 21.3, SD = 7.3). Regarding pharmacological treatment, 92.9% of participants were prescribed lithium, 57.1% levothyroxine, and 35.7% quetiapine. Polypharmacy was common (85.7%). Employment contexts were diverse, including students, healthcare workers and service sector roles.

**TABLE 1:** Baseline demographic characteristics of the patients and therapists.

Characteristic	*N* (%)
Participants (receiving intervention)	
Gender	
Male	4 (28.6)
Female	9 (64.3)
Prefer not to say	1 (7.1)
Marital status	
Single	11 (78.6)
Married	3 (21.4)
Education level	
Technical–professional	5 (35.7)
High school	5 (35.7)
University	4 (28.6)
Comorbidities	
Yes	8 (57.1)
No	6 (42.9)
Cigarette smoking	
Never	8 (57.1)
Current	4 (28.6)
Previous	2 (14.3)
Recreational drug use	
Never	9 (64.3)
Current	1 (7.1)
Previous	4 (28.6)
Previous psychotherapy	
Yes	11 (78.6)
No	3 (21.4)
Therapists	
Gender	
Male	2 (14.3)
Female	12 (85.7)
Country	
Chile	13 (92.9)
Spain	1 (7.1)
Graduate university	
Pontificia Universidad Catolica de Chile (UC)	2 (14.3)
Universidad de Valparaíso (UV)	9 (64.3)
Universidad de Santiago de Chile (USACH)	1 (7.1))
Pontificia Universidad Católica de Valparaíso (UCV)	1 (7.1)
Universidad de Deusto (Spain)	1 (7.1)
Professional background	
Clinical psychologists	14 (100)
Theoretical orientation	
Cognitive therapy	5 (35.7)
Narrative therapy	5 (35.7)
Systemic therapy	2 (14.3)
Humanistic therapy	1 (7.1)
Psychodynamic therapy	1 (7.1)
Postgraduate qualification	
Postgraduate certificate (PGCert)	10 (71.4)
Master's degree (M)	4 (28.6)

All therapists (*n* = 14) were licensed clinical psychologists actively delivering synchronous online therapy. They had completed a 5‐year psychology degree in Chile or Spain with supervised clinical training and held recognised postgraduate qualifications in psychotherapy (e.g., PGCert or Master's). Therapist mean age was 31.9 years (SD = 3.4), ranging from 27 to 38 years (see Table 1), with an average of 5.4 years of clinical experience (SD = 2.4) and 1.5 years of experience in telepsychology (SD = 0.5). Before participating in the study, all therapists received structured training in MCT through the official manual and e‐learning curriculum (www.uke.de/e‐mct), which was developed primarily for psychosis and designed for group delivery rather than being tailored to individual treatment or bipolar disorder. Training was delivered by the lead researcher, who has extensive experience implementing MCT in diverse clinical contexts.

### 2.3 Procedures

Therapists were enrolled first to ensure eligibility and logistical alignment, after which patient recruitment proceeded to enable matching with pre‐qualified therapists. Therapist–patient dyads were uniquely constituted, with each patient assigned to a single therapist, and matching was based on availability and scheduling compatibility using a convenience allocation procedure.

The lead psychiatrist of the research team, who was responsible for the clinical care of most participants, initially introduced the study to eligible patients during routine consultations. The lead researcher then verified inclusion and exclusion criteria, resolved any questions and obtained informed consent.

### 2.4 Intervention

In this study, MCT+ was adapted for individuals with BD‐I and translated into Chilean Spanish. This is the first known adaptation of MCT+ for Spanish‐speaking populations with affective disorders. The adaptation was formally authorised by the original author for use with Chilean patients.

Several modifications were made to ensure cultural and clinical relevance for the Chilean context. Linguistic adjustments adapted idiomatic expressions from Hispanic Spanish to Chilean Spanish. Given the focus on BD, terminology used in previous versions of MCT+ designed for nonaffective psychosis or schizophrenia was adjusted to better reflect mood dysregulation, manic and depressive symptoms and affective instability. Psychoeducational vignettes and exercises were updated to incorporate case material familiar to this clinical group, and culturally congruent examples were integrated to enhance engagement and comprehension. These adaptations were refined through consultation with local psychotherapists to ensure clinical and cultural appropriateness.

The intervention had 12 individual sessions (see Table S1), delivered weekly via Zoom Enterprise. Sessions followed the flexible and collaborative format of MCT+, whereby therapists could tailor the sequence and selection of modules to patient needs and clinical presentation. Screen‐sharing was used to present visual materials during sessions, and all modules included homework tasks to reinforce learning and generalisation. Both therapist and patient accessed the sessions through a permanent, secure link. All sessions were recorded, and participants were notified of this feature at the start of each meeting.

Therapy sessions were conducted entirely online between 2 September 2021 and 15 March 2023. Although this period corresponded to the latter stages of the COVID‐19 pandemic—when strict lockdowns were no longer in place—it reflected a broader global shift towards digital mental health provision, driven by residual public health restrictions and evolving service delivery models.

### 2.5 Measures

**FIGURE 1:**
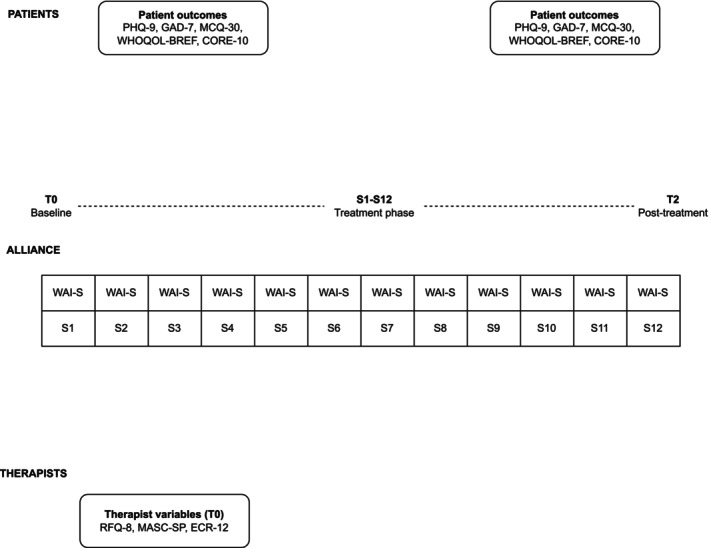
Temporal structure of assessments across the intervention. Therapist variables were assessed at baseline only (T0); patient outcomes were assessed at baseline (T0) and posttreatment (T2); and the therapeutic alliance was assessed after each therapy session (S1–S12). Note. WAI‐S = Working Alliance Inventory—Short Form; PHQ‐9 = Patient Health Questionnaire‐9; GAD‐7 = Generalised Anxiety Disorder‐7; CORE‐10 = Clinical Outcomes in Routine Evaluation—10‐item measure; MCQ‐30 = Metacognition Questionnaire‐30; WHOQOL‐BREF = World Health Organisation Quality of Life—BREF; RFQ‐8 = Reflective Functioning Questionnaire‐8; MASC‐SP = Movie for the Assessment of Social Cognition—Spanish version; ECR‐12 = Experiences in Close Relationships—12‐item version.

Measurement points are outlined in Figure 1. Therapist variables were assessed at baseline (T0) only; patient outcomes were assessed at baseline (T0) and posttreatment (T2); and the therapeutic alliance (WAI‐S) was assessed after each session (S1–S12).

#### 2.5.1 Therapist Measures

**Reflective Functioning Questionnaire‐8 (RFQ‐8)** (Fonagy et al. 2016) is a brief self‐report measure designed to assess dispositional tendencies in reflective functioning. It includes two subscales: RFQ_C (certainty about mental states), reflecting a rigid or hypermentalising style; and RFQ_U (uncertainty about mental states), associated with hypomentalising or difficulty understanding one's own and others' minds. Unlike performance‐based measures, RFQ‐8 captures subjective attitudes and habitual modes regarding mental states, rather than in‐the‐moment accuracy. The RFQ‐8 was completed at baseline by therapists to index their self‐perceived mentalising tendencies. The Spanish version (Ruiz‐Parra et al. 2023) has been validated in clinical populations retaining the original factor structure with adequate psychometric properties (*α* > 0.70). In the present sample, internal consistency was acceptable for therapists (*α* = 0.67 raw; *α* = 0.68 standardised; G6 = 0.88), indicating adequate reliability for a brief dispositional measure of reflective functioning.

**Movie for the Assessment of Social Cognition—Spanish Version (MASC‐SP)** (Dziobek et al. 2006). This 15‐min video‐based task assesses mentalising performance in complex social interactions through 43 forced‐choice items and yields both accuracy scores and error types (undermentalising, no Theory of Mind and hypermentalising). Although the task requires controlled processing, it is sensitive to variations along the automatic–controlled dimension of mentalising, based on the nature of inferences made. It was administered at baseline to therapists to assess mentalisation under dynamic and ecologically valid conditions. The Spanish version (Lahera et al. 2014) demonstrates strong psychometric properties (*α* = 0.86).

**Experiences in Close Relationships—12 Item version (ECR‐12)** (Wei et al. 2007). The ECR‐12 is a 12‐item measure assessing adult attachment avoidance and anxiety. The Chilean adaptation (Spencer et al. 2013) has strong psychometric properties, including internal consistency (*α* = 0.80–0.83) and construct validity. Therapists completed the scale at baseline. In the present sample, reliability was excellent for patients (*α* = 0.94, G6 = 1.00) and good for therapists (*α* = 0.82, G6 = 0.97).

#### 2.5.2 Participant Measures

**Patient Health Questionnaire‐9 (PHQ‐9)** (Kroenke et al. 2001) is a widely used self‐report measure of depressive symptoms. It has strong psychometric properties, including high internal consistency (*α* = 0.86–0.89), robust test–retest reliability (*r* = 0.84–0.95) and well‐established construct and criterion validity (Kroenke et al. 2001; Löwe et al. 2004; Martin et al. 2006; Titov et al. 2011). The PHQ‐9 has been extensively validated in South American contexts, including Chile (Baader et al. 2012), with high internal consistency (*α* = 0.90) and good diagnostic accuracy (Errazuriz et al. 2022). In the present sample, internal consistency was excellent at both time points (PRE *α* = 0.93, G6 = 0.97; POST *α* = 0.96, G6 = 0.99).

**Generalised Anxiety Disorder‐7 (GAD‐7)** is a 7‐item self‐report instrument assessing symptoms of generalised anxiety. It was administered at baseline and posttreatment. The measure demonstrates strong internal consistency (*α* = 0.89–0.92) and good criterion validity. The Spanish version (García‐Campayo et al. 2010) has shown adequate psychometric properties and clinical utility. In the present sample, reliability was excellent (PRE: *α* = 0.92, G6 = 0.94; POST: *α* = 0.96, G6 = 0.99).

**World Health Organisation Quality of Life—Brief Version (WHOQOL‐BREF)** (WHOQOL Group 1998) is a 26‐item self‐report measure of perceived quality of life across four domains (physical, psychological, social and environmental), administered at baseline and posttreatment. It has been validated in Spanish‐speaking populations and shows strong internal consistency and construct validity. In this study, reliability was good (PRE *α* = 0.93, G6 = 0.90; POST *α* = 0.96, G6 = 0.92).

**Clinical Outcomes in Routine Evaluation—10 (CORE‐10)**(Barkham et al. 2013) The CORE‐10 is a 10‐item self‐report scale measuring global psychological distress and monitoring therapeutic change. It is derived from the longer CORE‐OM and maintains strong psychometric properties, with reported internal consistency coefficients typically above *α* = 0.85 and good test–retest reliability (Valdiviezo‐Oña et al. 2024). Items are rated on a 5‐point Likert scale. In this study, reliability was high at both pre‐ and posttreatment (PRE: *α* = 0.88, G6 = 0.95; POST: *α* = 0.90, G6 = 0.93).

**Working Alliance Inventory–Short Form (WAI‐S)** (Horvath and Greenberg 1989), an extensively validated 12‐item self‐report scale assessing the therapeutic alliance across three dimensions: Goal, Task and Bond. The Spanish version (Andrade‐González and Fernández‐Liria 2016) demonstrates high internal consistency (*α* > 0.85) and replicates the original factorial structure. Patients completed the WAI‐S after each therapy session to track alliance development over time. In the present study, reliability was strong across key time points (Session 1: *α* = 0.88, G6 = 1.00; Session 4: *α* = 0.90, G6 = 0.92; Session 12: *α* = 0.85, G6 = 0.92).

**Metacognitive Questionnaire‐30 (MCQ‐30)** (Wells and Cartwright‐Hatton 2004) assesses self‐reported metacognitive beliefs, completed by patients at baseline and posttreatment. It has 30 items and five subscales: Positive Beliefs about Worry, Negative Beliefs about Uncontrollability and Danger, Cognitive Confidence, Need to Control Thoughts and Cognitive Self‐Consciousness. The Spanish adaptation (Ramos‐Cejudo et al. 2013) used in this study has shown excellent internal consistency across all subscales (*α* = 0.80–0.88) and strong construct validity in clinical populations. In the present sample, internal consistency was satisfactory (PRE *α* = 0.76, G6 = 0.94; POST *α* = 0.76, G6 = 0.94).

### 2.6 Ethics

Favourable ethical opinion was received from the Research Ethics Committee (REC) of the School of Health in Social Science, University of Edinburgh, and from the Chilean Ethics Committee (CEC) of the Valparaíso‐San Antonio Health Service.

### 2.7 Data Analysis

To evaluate pre–posttreatment effects, paired *t*‐tests or Wilcoxon signed‐rank tests were conducted on patient outcomes (GAD‐7, PHQ‐9, WHOQOL‐BREF, MCQ‐30 and CORE‐10). Two‐tailed tests were used, with normality assessed via Shapiro–Wilk tests. Effect sizes (Cohen's *d*) were computed for each measure to estimate the magnitude of pre–postchange and facilitate interpretation of clinical relevance (Cohen 1977). Where distributional assumptions were uncertain, nonparametric sensitivity analyses were used to support robustness in this small sample, as these approaches do not rely on strict normality assumptions. Reliable Change Indexes (RCIs) were calculated to identify cases of reliable improvement or deterioration at the individual level (Jacobson and Truax 1991), providing complementary information on individual‐level change beyond group statistics. Associations between therapist‐level variables (mentalisation and attachment) and the therapeutic alliance were explored using Spearman's rank correlation coefficients. Given the small sample and lack of normality, nonparametric correlations were preferred.

To address whether therapist mentalisation capacity influenced the development of the therapeutic alliance over time—a multilevel modelling approach was applied using hierarchical linear modelling (HLM; see Figure S1) following a stepwise approach. HLM allows for simultaneous modelling of within‐person change (Level 1) and between‐person predictors (Level 2), such as therapist characteristics, and can account for nested data structures (repeated WAI‐S measures nested within dyads), unbalanced data, and variable session attendance. Given the small number of therapist–patient dyads (*n* = 14) and variability in observations per dyad, multilevel model estimates were interpreted as preliminary and hypothesis‐generating, consistent with prior discussions of sample size considerations in multilevel modelling (Gallop and Tasca 2009; Tasca and Gallop 2009). First, a random‐intercept model (Model 1) assessed baseline alliance variability across dyads. Session number was added as a fixed effect and then as a random slope (Model 2) to model individual change trajectories. An autoregressive structure (AR1) was specified for residuals to account for temporal dependency across repeated measures (Model 3). Therapist MASC‐SP scores were introduced as a Level‐2 fixed effect (Model 4), followed by RFQ‐8 scores (Model 5). Lastly, an interaction term between MASC and RFQ (Model 6) tested the hypothesis that flexible switching between automatic and reflective modes of mentalising (i.e., the *mentalisation switch*) enhances alliance development. All models were estimated using the *lmer* function from the *lme4* package in R (Bates et al. 2015), except for the model including autoregressive residuals (AR[1]), which was fitted using the *lme* function from the *nlme* package (Pinheiro et al. 2023). All models were estimated using restricted maximum likelihood (REML).

## 3 Results

### 3.1 Effectiveness of MCT+

Pre‐ and posttreatment scores were compared across standardised measures to evaluate symptomatic and cognitive change (see Table 2). Participants showed significant reductions in anxiety symptoms (*t*(13) = 3.42, *p* = 0.005, *d* = 0.64, moderate effect) and psychological distress (*t*(13) = 2.98, *p* = 0.008, *d* = 0.59, moderate effect), as well as a modest improvement in metacognitive beliefs (*t*(13) = 1.56, *p* = 0.14, *d* = 0.37, small–moderate effect). Depression (*t*(13) = 1.21, *p* = 0.24, *d* = 0.34, small effect) and quality of life (*t*(13) = 0.88, *p* = 0.39, *d* = 0.21, negligible effect) did not show significant change. Notably, the MCQ‐30 Cognitive Self‐Consciousness subscale decreased (*p* = 0.026), suggesting reduced overmonitoring of thoughts.

**TABLE 2:** Pre–postchanges in mean scores for participant outcomes.

Measure	Pretreatment mean (SD)	Posttreatment mean (SD)	*p*
PHQ‐9	11.00 (5.40)	8.14 (4.90)	0.087
GAD‐7	10.08 (4.10)	5.75 (4.30)	0.032*
CORE‐10	1.16 (0.68)	0.73 (0.72)	0.008**
MCQ‐30 global	71.57 (9.50)	63.07 (10.20)	0.049*
MCQ‐30 positive beliefs	12.50 (3.20)	10.21 (4.10)	0.063
MCQ‐30 negative beliefs	15.21 (5.10)	13.50 (5.80)	0.238
MCQ‐30 cognitive confidence	14.93 (3.70)	14.21 (4.20)	0.428
MCQ‐30 need for control	12.21 (4.80)	10.86 (4.90)	0.194
MCQ‐30 cognitive S‐C	16.71 (3.80)	14.29 (4.50)	0.026*
WHOQOL‐BREF overall	15.17 (3.60)	15.50 (3.40)	0.791
WHOQOL‐BREF physical health	14.43 (3.60)	15.24 (3.40)	0.477
WHOQOL‐BREF psychological health	14.22 (3.60)	14.78 (3.40)	0.392
WHOQOL‐BREF social relationships	13.78 (3.60)	14.00 (3.40)	1.000
WHOQOL‐BREF environment	16.12 (3.60)	16.42 (3.40)	0.844

Abbreviation: SD = standard deviation.

* = *p* < 0.05.

** = *p* < 0.01.

Effect‐size estimates indicate that digital MCT+ produced clinically meaningful reductions in anxiety and distress, with smaller gains in metacognitive beliefs. In addition to group‐level effects, RCI analyses highlighted heterogeneity in individual clinical change, with a subset of participants showing reliable improvement and isolated cases of deterioration. At the individual level, RCI analyses supported these findings: most participants remained within the nonsignificant range, with isolated cases of reliable improvement or deterioration (see Figures S1–S6).

### 3.2 Therapist Characteristics and Therapeutic Alliance

Table 3 presents the correlations between therapist characteristics and the dimensions of the Working Alliance Inventory (WAI). For the RFQ, a moderate unadjusted positive correlation was observed between reflective functioning and the *Task* subscale (rho = 0.69, *p* = 0.012), although this became nonsignificant after Holm adjustment. No statistically significant associations were observed between attachment style and any WAI dimension.

**TABLE 3:** Correlations between therapist characteristics and WAI dimensions.

Therapist characteristic	WAI dimension	*ρ* (Unadjusted)	*p* (Unadjusted)	*ρ* (Adjusted)	*p* (Adjusted)
ECR (Attachment)	Bond	−0.54	0.073	−0.54	> 0.999
	Goals	0.25	0.436	0.25	> 0.999
	Task	0.16	0.624	0.16	> 0.999
	Overall WAI	−0.05	0.880	−0.05	> 0.999
RFQ (Mentalisation)	Bond	0.40	0.202	0.40	> 0.999
	Goals	0.54	0.069	0.54	> 0.999
	Task	0.69	0.*012*	0.69	> 0.999
	Overall WAI	0.51	0.091	0.51	> 0.999
MASC (Mentalisation)	Bond	−0.16	0.627	−0.16	> 0.999
	Goals	0.14	0.662	0.14	> 0.999
	Task	0.26	0.422	0.26	> 0.999
	Overall WAI	0.02	0.939	0.02	> 0.999

*Note:* Spearman's rho (*ρ*) coefficients. Adjusted *p*‐values computed using Holm correction for multiple comparisons. *p* < 0.05 (uncorrected).

### 3.3 How Does Therapist Mentalisation Capacity Affect Development of the Therapeutic Alliance During Digital MCT +?

Results of the HLM are displayed in Tables 4 and 5. Across all models, session progression did not significantly predict total WAI scores, indicating that the therapeutic alliance did not improve or decline in a consistent linear fashion across time. However, the Bond subscale exhibited a distinct trajectory. Session‐level improvements in Bond reached statistical significance in multiple models (Model 3: *b* = 0.17, *p* = 0.005; Model 4: *b* = 0.17, *p* = 0.014; Model 5: *b* = 0.16, *p* = 0.022), with moderate conditional *R* ^2^ values ranging from 0.723 to 0.764, indicating both meaningful within‐patient variability and substantial between‐dyad differences. The addition of therapist‐level predictors incrementally improved model fit. In Model 6, both therapist MASC and RFQ emerged as significant individual predictors of Bond (MASC: *b* = 4.61, *p* = 0.011; RFQ: *b* = 7.22, *p* = 0.009), with a significant, negative interaction (*b* = −0.19, *p* = 0.011), suggesting that therapists with simultaneously high scores on both automatic (MASC) and reflective (RFQ) mentalising were associated with weaker bonds with patients. This implies that an overreliance on both intuitive and self‐conscious mentalising processes may be associated with reduced flexibility in responding to patient cues.

**TABLE 4:** Summary of mixed‐effects models for the overall therapeutic alliance.

Predictor	Model 1	Model 2	Model 3	Model 4	Model 5	Model 6
Intercept	72.39 (< 0.001***)	70.12 (< 0.001***)	69.97 (< 0.001***)	54.63 (0.016*)	30.38 (0.301)	−365.56 (0.013*)
Session	—	0.38 (0.109)	0.43 (0.072)	0.37 (0.114)	0.34 (0.144)	0.34 (0.130)
MASC (Therapist)	—	—	—	0.43 (0.433)	0.59 (0.312)	11.41 (0.006**)
RFQ‐8 (Therapist)	—	—	—	—	0.80 (0.220)	17.21 (0.006**)
MASC × RFQ interaction	—	—	—	—	—	−0.45 (0.008**)
AIC	1089.25	1079.53	1071.70	1080.34	1080.26	1075.96
BIC	1098.45	1097.94	1093.18	1101.83	1104.81	1103.58
*R* ^2^ (conditional)	0.654	0.772	0.736	0.787	0.769	0.746
RMSE	6.19	5.52	5.74	5.52	5.49	5.51
Log‐likelihood	−541.62	−533.76	−528.85	−533.17	−532.13	−528.98

*Note:* Hierarchical linear models examining the effect of therapist mentalisation capacity (MASC, RFQ‐8) and their interaction on the total therapeutic alliance (WAI). Coefficients represent fixed effects estimates with corresponding *p*‐values in parentheses. All models included random intercepts for participants. Model 6 includes the interaction between therapist MASC and RFQ scores. Conditional *R* ^2^ reflects explained variance including both fixed and random effects. Values in parentheses are *p*‐values. Significance levels.

* *p* < 0.05.

** *p* < 0.01.

*** *p* < 0.001.

**TABLE 5:** Summary of mixed‐effects models for working alliance bonding subscale.

Predictor	Model 1	Model 2	Model 3	Model 4	Model 5	Model 6
Intercept	23.62 (*p* < 0.001) ***	22.61 (*p* < 0.001) ***	22.61 (*p* < 0.001) ***	20.49 (*p* = 0.028) *	11.92 (*p* = 0.341)	−154.22 (*p* = 0.017) *
Session	—	0.17 (*p* = 0.013) *	0.17 (*p* = 0.005) **	0.17 (*p* = 0.014) *	0.16 (*p* = 0.022) *	0.15 (*p* = 0.029) *
MASC (Therapist)	—	—	—	0.06 (*p* = 0.799)	0.10 (*p* = 0.694)	4.61 (*p* = 0.011) *
RFQ‐8 (Therapist)	—	—	—	—	0.32 (*p* = 0.262)	7.22 (*p* = 0.009) **
MASC × RFQ (Interaction)	—	—	—	—	—	−0.19 (*p* = 0.011) *
AIC	764.75	763.88	762.18	767.00	769.01	767.50
BIC	773.95	782.29	783.66	788.48	793.56	795.12
*R* ^2^ (conditional)	0.715	0.747	0.723	0.764	0.762	0.743
RMSE	2.19	2.08	2.11	2.08	2.07	2.07
Log‐likelihood	−379.37	−375.94	−374.09	−376.50	−376.50	−374.75

**FIGURE 2:**
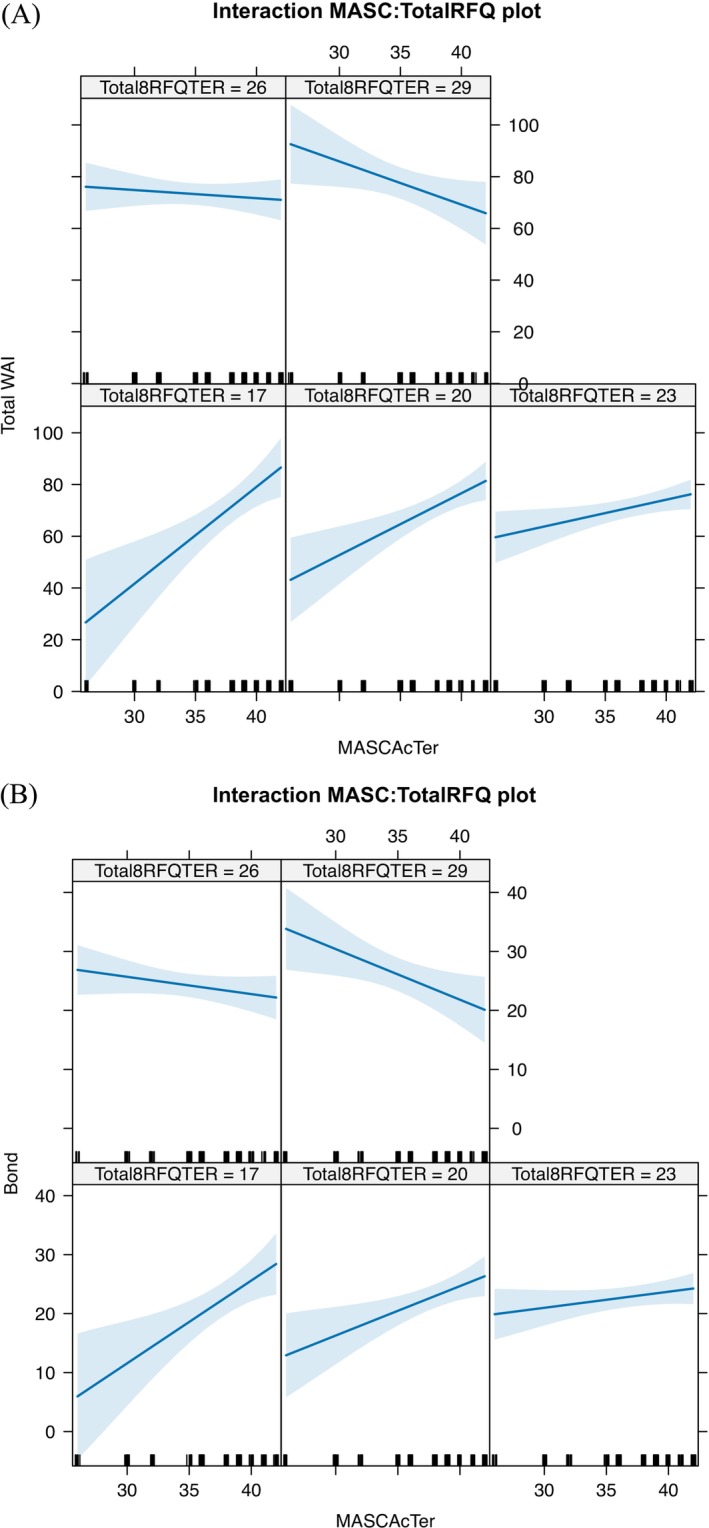
Interaction between therapist automatic and reflective mentalising capacities in predicting working alliance. (A) Interactions between Mentalising and Working Alliance Total Score. *Note*: Interaction effects between therapists' automatic mentalising (MASC) and reflective functioning (RFQ‐8) on the overall therapeutic alliance (WAI Total). Each panel represents predicted WAI scores across MASC values at specific RFQ levels (17, 20, 23, 26 and 29). Blue lines indicate modelled trends; shaded areas represent 95% confidence intervals. At lower RFQ levels, higher MASC is associated with stronger alliance scores, while at higher RFQ levels, this relationship reverses, suggesting a nuanced interplay between automatic and reflective mentalising capacities in shaping alliance quality. (B) Interactions between Mentalising and Working Alliance Bonding Subscale Note. Each panel represents a different RFQ level (17, 20, 23, 26 and 29), allowing visual exploration of how the relationship between MASC scores and the Bond subscale varies with therapist reflective functioning. At low RFQ levels, higher MASC scores are associated with stronger Bond ratings, suggesting that automatic mentalising supports therapeutic connection when reflective functioning is limited. Conversely, at high RFQ levels, the association reverses, indicating that an overreliance on mentalising—both automatic and reflective—may interfere with emotional closeness in therapy.

Further examination of the interaction revealed phase‐specific patterns (see Figure 2A *)*. In early sessions, higher MASC combined with lower RFQ predicted stronger Bond scores, highlighting the value of automatic mentalisation for establishing emotional connection when reflective processes may still be emerging. As therapy progressed, higher RFQ became increasingly predictive of the Task and Goal subdomains, suggesting a sequential or phase‐specific deployment of mentalising capacities. In Model 5 therapist mentalisation variables (MASC, RFQ) did not significantly predict total WAI scores when entered alone but *t*he MASCxRFQ interaction (Model 6) significantly accounted for between‐dyad variance and improved model fit indices (*b* = −0.45, *p* = 0.008, AIC = 1075.96; BIC = 1103.58). This suggests that mentalising flexibility—rather than mentalising capacity alone—is implicated in alliance development in digital MCT+.

The Bond dimension of the therapeutic alliance emerged as the most responsive to within‐session change, with moderate intraclass correlation coefficients (ICCs) (range = 0.715–0.758) reflecting both between‐ and within‐dyad variability. The interaction between therapist MASC and RFQ scores revealed a non‐linear effect (see Figure 2B). At *low RFQ*, higher automatic mentalising (MASC) predicted greater bonding, while at *high RFQ*, increased MASC was associated with weaker alliance, indicating that therapists with low dispositional reflection may have leveraged intuitive mentalising to build emotional connection, whereas overreliance on both systems may have reduced relational attunement. In contrast, Goals and Tasks subscales demonstrated limited within‐session variability, with nonsignificant session effects and high ICCs indicating substantial between‐dyad stability.

## 4 Discussion

The current study suggested mixed effectiveness for digitally delivered MCT+ in individuals with BD‐I. After 12 weeks, significant improvements were observed in anxiety, general psychological distress and global metacognitive beliefs, indicating that the intervention targeted maladaptive metacognitive patterns and was associated with symptom relief in specific cognitive–emotional domains. However, depressive symptoms, quality of life and specific metacognitive subscales did not show significant change.

Effect‐size estimates (Cohen 1977) showed moderate reductions in anxiety and psychological distress, and small‐to‐moderate improvements in metacognitive beliefs—patterns consistent with prior evidence that MCT primarily modifies cognitive style and anxiety‐related processes in severe mental illness. By contrast, the absence of significant change in depressive symptoms or quality of life supports previous findings that mood stability and global functioning often require longer or more integrative therapeutic approaches, suggesting that MCT+ may be best positioned as a complementary rather than stand‐alone intervention (Chatterton et al. 2017; de Siqueira Rotenberg et al. 2025; Goes 2023; Haffner et al. 2018). The overall pattern of pre–postfindings was supported by sensitivity analyses using nonparametric tests, increasing confidence that results were not driven by distributional violations in this small sample. Given the exploratory case‐series design and modest effect sizes, these findings are best interpreted as preliminary indicators of feasibility and potential mechanisms rather than as causal evidence.

Anxiety in individuals with BP is correlated with increased impairment and relapse (Pavlova et al. 2015; Yapici Eser et al. 2018). Despite some evidence for cognitive behavioural therapies (CBT) approaches tailored to anxiety in BP (Seeberg et al. 2021; Stratford et al. 2015), few interventions prioritise anxiety as a primary target. In our study, MCT+ was associated with reductions in anxiety via a putative metacognitive mechanism, namely, the targeting of threat appraisals and perseverative thinking. This is consistent with theory and MCT technique (Leanza et al. 2020; Meinhart et al. 2025; Moritz et al. 2018; Schneider et al. 2023), positioning MCT+ as an adjunctive intervention in multimodal care.

Conversely, the null findings for depressive symptoms reflect a broader picture of modest, inconsistent effects for psychological treatments for bipolar depression, particularly brief format approaches (Salcedo et al. 2016; Yilmaz et al. 2022). Given MCT+'s focus on cognitive style rather than affective content, it may lack specificity to shift chronic depressive states. Future adaptations may therefore benefit from phase‐sensitive modules or integration within longer term frameworks.

### 4.1 Mentalisation as a Dynamic Driver of Therapeutic Alliance in Digital MCT

For therapist mentalisation, a large proportion of alliance variance was attributable to between‐patient differences, suggesting a trait‐like influence of therapist capacity. Surprisingly, high scores on both the MASC and RFQ predicted weaker alliances, indicating that overactivation of both implicit and explicit mentalisation may reduce therapeutic attunement. Therapists with higher MASC and lower RFQ formed stronger early alliances, possibly due to the benefits of intuitive, flexible engagement. Bond subdimension scores improved over time while Task and Goal subdimensions remained stable, distinct phase‐specific mentalising demands.

The high ICC values suggest that these mentalising traits were consistently expressed across sessions. However, the significant RFQ × MASC interaction indicates that alliance development depended less on overall capacity than on dynamic balance. In digital formats, where non‐verbal cues are attenuated, this balance may be particularly delicate. Reduced access to embodied and affective signals may prompt greater reliance on verbal and reflective inference, with a risk of overcompensation if not flexibly regulated. In individuals with BD, who commonly exhibit fluctuations in affect, interpersonal trust and metacognitive coherence (Sperry and Kwapil 2020, 2019), these demands place additional strain on therapist mentalising capacity.

Digital delivery further attenuates embodied cues essential for intuitive mentalisation. In videoconferencing, non‐verbal feedback loops—such as eye gaze, body posture and microexpressions—are often diminished, leading to greater reliance on reflective, verbalised inference. This may inadvertently tip therapists into teleological or pretend modes of mentalisation (Bateman and Fonagy 2019), thereby disconnecting interpretations from the patient's immediate experience. Accordingly, the observed RFQ × MASC interaction can be conceptualised as a compensatory process: When intuitive inference is constrained, reflective mentalisation may upregulate to maintain engagement. Nevertheless, MASC and RFQ are best understood as trait‐level indicators approximating automatic and reflective mentalising tendencies, rather than process–pure measures of in‐session mentalisation.

Use of a structured, metacognitively oriented intervention such as MCT+ offers further insights. MCT+ requires therapists to navigate a hybrid task: manualised scaffolding gives a stable, predictable structure, facilitates consistency and reduces ambiguity within the dyad. However, it also demands moment‐to‐moment mentalising flexibility, adapting the delivery of structured modules to the patient's shifting mood, cognition and epistemic stance.

In this model, therapist alliance can be reconceptualised as a broader regulatory mechanism that balances structure with responsiveness, enabling therapists to switch between mentalising modes in a phase‐sensitive manner. Within the bipolar‐digital‐MCT+ triad, therapists must recalibrate their trait capacities to the fluctuating demands of therapy. Therapeutic effectiveness in such settings therefore depends on overall mentalising ability, alongside the capacity to contextually sequence and modulate these skills.

### 4.2 Therapist Mentalisation and the Effectiveness of Brief MCT+

While alliance strength itself was not significantly correlated with symptom outcomes, its role as a mediating or moderating factor remains plausible. The dynamic interaction between RFQ and MASC was associated with *phase‐specific shifts* in alliance dimensions, suggesting that therapists' ability to flexibly alternate between automatic and controlled mentalisation may have shaped the therapeutic process in ways not directly captured by linear symptom‐outcome models.

Reliable change index findings further highlighted marked individual variability in therapeutic gains. Despite significant group‐level reductions in anxiety and distress, not all patients achieved reliable clinical change, underscoring heterogeneity in response and the likelihood of subgroup‐level benefit. This variability may partly reflect differential therapist capacities to sustain mentalising across clinical presentations. For example, therapists with higher RFQ but lower MASC may have effectively supported structured metacognitive tasks, contributing to improvements in global metacognitive beliefs, while struggling to attune affectively in real time. Conversely, therapists with stronger MASC scores may have facilitated early engagement and emotional connection, supporting reductions in anxiety and general distress. These interpretations align with the finding that anxiety—but not depression—showed consistent pre–post and RCI‐level improvements.

One possibility is that the combination of a highly structured intervention format and digital delivery may have privileged cognitive over affective change. It may also have constrained intuitive modes of engagement (MASC), reducing opportunities for embodied coregulation even for highly skilled therapists.

### 4.3 The Mentalisation Switch as a Clinical Mechanism

The significant interaction between implicit (MASC) and explicit (RFQ) mentalising capacities also offers preliminary empirical support for a dynamic regulatory process best conceptualised as a *mentalisation switch* (Fonagy et al. 2011; Fonagy and Adshead 2012). Rather than acting as additive strengths, automatic and reflective mentalisation exerts contextually opposing effects on alliance development when coactivated at high levels. Therapists with elevated scores on both dimensions may therefore struggle to modulate their responses adaptively, becoming rigid or overanalytical.

Our findings highlight the importance of flexibility over the magnitude of mentalisation. Therapists with high MASC and lower RFQ appeared particularly effective in establishing early rapport, whereas RFQ became more relevant in later sessions, supporting task alignment and collaborative goal setting. This temporal differentiation reinforces the need for phase‐sensitive deployment of mentalising modes.

Within this framework, the *mentalisation switch* could denote the therapist's capacity to regulate when and how to deploy different forms of mentalisation in accordance with the relational and emotional demands of therapy. Rather than representing fixed traits, MASC and RFQ may operate as latent strategies deployed in treatment through sequencing and contextual responsiveness. When this modulation fails—e.g., when therapists rely simultaneously and excessively on both systems—mentalisation may become congested or fragmented, undermining the alliance. This model of dynamic switching is supported by the variability observed in our data. The high ICCs suggest that therapist traits explained a substantial portion of alliance variance across patients, yet the moderate conditional *R* ^2^ values in the Bond indicate that within‐dyad variation remained meaningful.

These residual fluctuations may reflect moments when the therapist's ability to switch between modes was either successful (e.g., adapting style in response to the patient's epistemic state) or unsuccessful (e.g., overrelying on deliberation in emotionally charged moments). However, given the small number of dyads and unbalanced session attendance, these nested‐model findings should be considered preliminary and warrant replication; future studies should also conduct systematic residual diagnostics to evaluate model assumptions and strengthen inference regarding the appropriateness of multilevel estimation.

### 4.4 Clinical Implications

These findings have several implications for clinical training and reflective practice. Clinically, the *mentalisation switch* may be observed in small yet meaningful shifts in the therapist's stance. For instance, emotionally attuned silence or validation during hypomanic speech may reflect automatic mentalisation, whereas subsequent collaborative goal setting may require reflective processing. The interaction between RFQ and MASC also suggests that effective therapists are not simply ‘high mentalisers’, but adaptive ones—those who can deploy intuitive attunement when necessary and shift into reflective processing to manage complexity or regulate ruptures.

This versatility is particularly critical in teletherapy, where diminished intuitive cues may increase reliance on verbalised reflection and risk disconnection. Supervisors should therefore attend not only to therapists' reflective capacity but also to the timing and contextual appropriateness of different mentalising modes. RFQ and MASC may serve as useful developmental tools within training and supervision, supporting targeted enhancement of intuitive attunement or reflective articulation as needed.

### 4.5 Limitations and Future Directions

Several limitations should be noted. First, the small sample size and the use of multiple statistical tests increase the risk of Type I error, warranting cautious interpretation of all associations. Purposive sampling and exclusion of individuals with severe symptoms or limited digital access may restrict generalisability to more complex or underserved populations (Borghouts et al. 2021). Second, therapist mentalisation was assessed exclusively via trait‐based measures at baseline. Although these instruments capture dispositional tendencies, they do not reflect moment‐to‐moment fluctuations in in‐session mentalisation (Luyten et al. 2024). Furthermore, the RFQ is a self‐report measure and therefore susceptible to social desirability bias, while the MASC—developed to detect subtle deficits—may show reduced sensitivity in high‐functioning samples. Ceiling effects may thus have limited discrimination between therapists.

Moreover, all therapists were relatively young and early in their clinical careers, with broadly similar training backgrounds, which may limit the generalisability of findings to more experienced or differently trained practitioners.

Notwithstanding these constraints and the exploratory nature of the design, the study provides preliminary and methodologically rigorous evidence regarding relational mechanisms in remote MCT+. Future research should examine these processes in larger and more diverse samples, incorporate time‐sensitive measures of therapist mentalisation and refine digital platforms to better support embodied and interpersonal signalling. Longitudinal designs will be essential to clarify how therapist mentalising capacities evolve across treatment and interact dynamically with patient characteristics to shape alliance and outcomes over time.

## Author Contributions

**Roberto Maluenda‐Gatica** contributed to conceptualisation, methodology, investigation, formal analysis, data curation, writing – original draft, visualisation and project administration and coordinated the overall research process and participant engagement. **Angus MacBeth** contributed to supervision, methodology and writing – review and editing. **Christian Araya** contributed to methodology, formal analysis and validation, particularly in relation to hierarchical linear modelling, and to writing – review and editing. **Javier Morán‐Kneer** and **Ulises Ríos** contributed to resources and investigation by facilitating access to the clinical sample and to writing – review and editing. **Steffen Moritz** contributed to conceptualisation, methodology and writing – review and editing and authorised the use of the MCT+ protocol.

## Funding

This work was supported by the National Agency for Research and Development (ANID), Doctorado Becas Chile Programme, awarded to the first author. The funders had no role in the study design, data collection, analysis, interpretation or publication.

## Conflicts of Interest

Steffen Moritz is one of the developers of Metacognitive Training (MCT). He declares no financial interests related to the present study, and the intervention was implemented independently by the research team. All analyses and interpretations were conducted without his involvement in data handling or statistical evaluation. The other authors declare no conflicts of interest.

## Supporting information


**Figure S1:** Structure of the hierarchical linear model (HLM).
**Figure S2:** Reliable and Clinically Significant Change (RCI) Classification for PHQ‐9 Depression Scores.
**Figure S3:** Reliable and Clinically Significant Change (RCI) Classification for GAD‐7 Anxiety Scores.
**Figure S4:** Reliable and Clinically Significant Change (RCI) Classification for CORE‐10 Psychological Distress.
**Figure S5:** Reliable and Clinically Significant Change (RCI) Classification for WHOQOL‐BREF Quality of Life.
**Figure S6:** Reliable and Clinically Significant Change (RCI) Classification for MCQ‐30 Metacognitive Beliefs.
**Table S1:** Session‐by‐session MCT+ protocol for individual participants.

## Data Availability

The data that support the findings of this study are available from the corresponding author upon reasonable request.
