# Supplementary material for: The Mentalisation Switch: Therapist Reflective Capacity and Alliance Dynamics in Digital MCT+ for Bipolar Disorder—A Longitudinal Quantitative Case Series
Source: Clin Psychol Psychother. 2026 Mar 19;33(2):e70260. doi: 10.1002/cpp.70260 (PMC13001804; doi:10.1002/cpp.70260)
Supplement: Supplementary file 1 — Figure S1: Structure of the hierarchical linear model (HLM). Figure S2: Reliable and Clinically Significant Change (RCI) Classification for PHQ‐9 Depression Scores. Figure S3: Reliable and Clinically Significant Change (RCI) Classification for GAD‐7 Anxiety Scores. Figure S4: Reliable and Clinically Significant Change (RCI) Classification for CORE‐10 Psychological Distress. Figure S5: Reliable and Clinically Significant Change (RCI) Classification for WHOQOL‐BREF Quality of Life. Figure S6: Reliable and Clinically Significant Change (RCI) Classification for MCQ‐30 Metacognitive Beliefs. Table S1: Session‐by‐session MCT+ protocol for individual participants. [file CPP-33-e70260-s001.docx]

**Supplementary Material**

**Supplementary Figure 1. Structure of the Hierarchical Linear Model (HLM).**


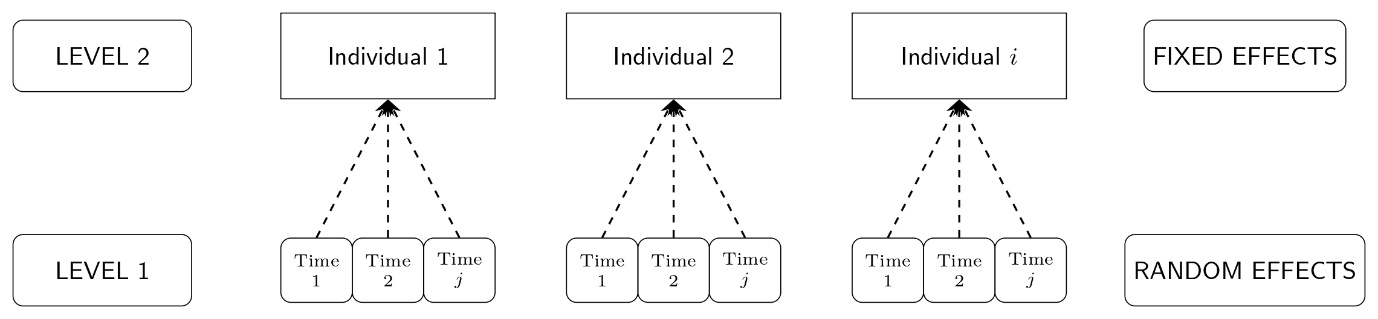


**Note.** This diagram illustrates the two-level data structure modelled in the analysis of session-by-session therapeutic alliance. At **Level 1**, repeated measurements (e.g., alliance ratings) were collected at multiple time points (Time 1 to Time j) for each individual, capturing **within-person change over time**. These time points are shown as **nested within individuals** through dashed arrows. At **Level 2**, each individual (or dyad) represents a higher-level unit, accounting for **between-person variability**, such as differences in therapist mentalising capacity or baseline alliance. This hierarchical structure allows for the estimation of both **fixed effects** (shared across the sample) and **random effects** (individual-specific variation), offering a nuanced understanding of how the therapeutic alliance evolves across sessions while considering person-level predictors.

**Supplementary Figure 2**. Reliable and Clinically Significant Change (RCI) Classification for PHQ-9 Depression Scores.


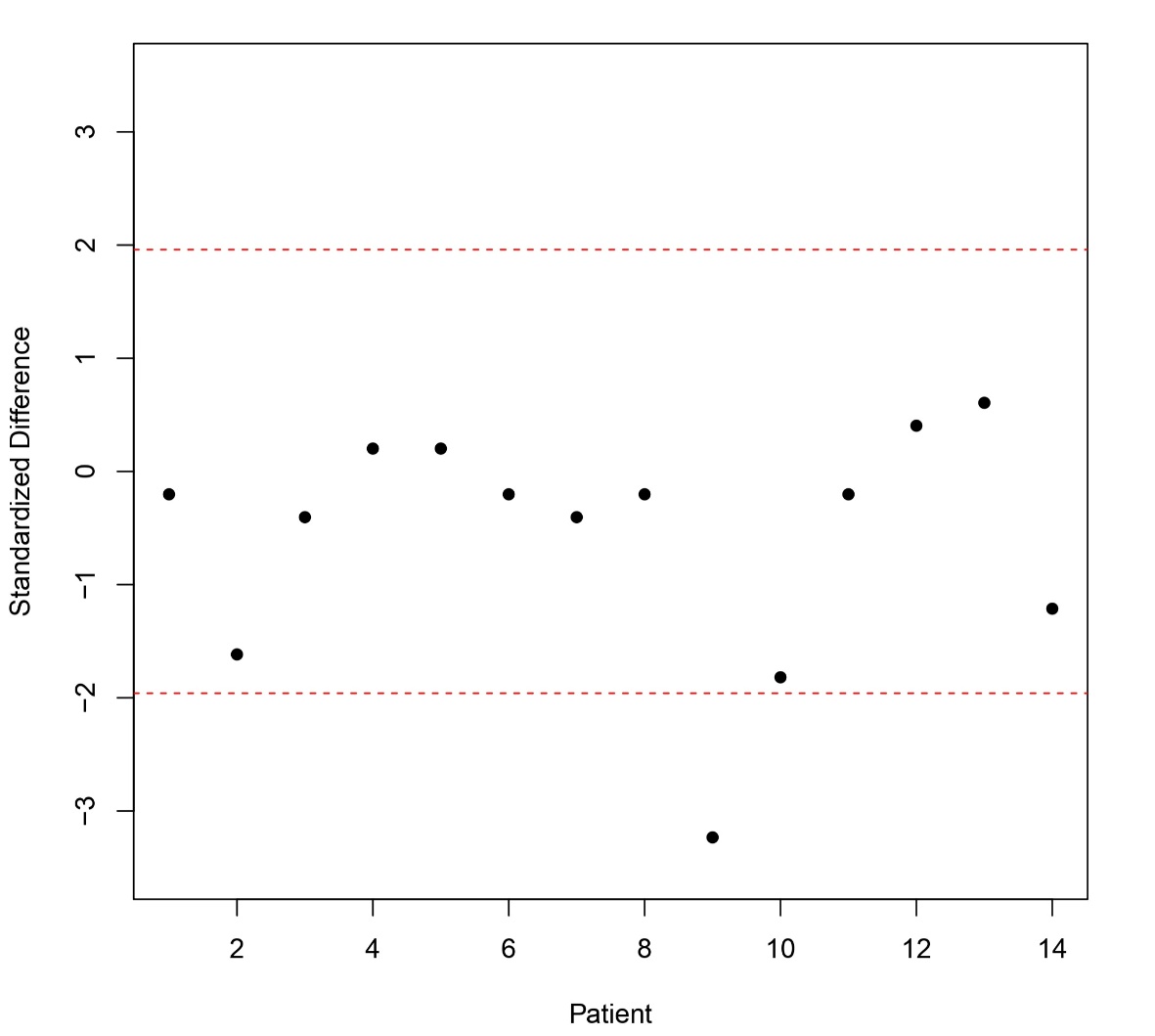


**Note.** Reliable Change Index (RCI) values for PHQ-9 scores ranged from −1.62 (Patient 2) to +0.60 (Patient 13), indicating minor fluctuations and overall stability in depressive symptoms for most participants following the intervention. One participant (Patient 9) showed a reliable deterioration (RCI = −3.23), while another (Patient 10) demonstrated a negative trend approaching the threshold for reliable change (RCI = −1.82) but not meeting the criterion. These findings suggest largely stable depressive symptom levels, with isolated cases of potential worsening requiring further investigation.

**Supplementary Figure 3.** Reliable and Clinically Significant Change (RCI) Classification for GAD-7 Anxiety Scores.


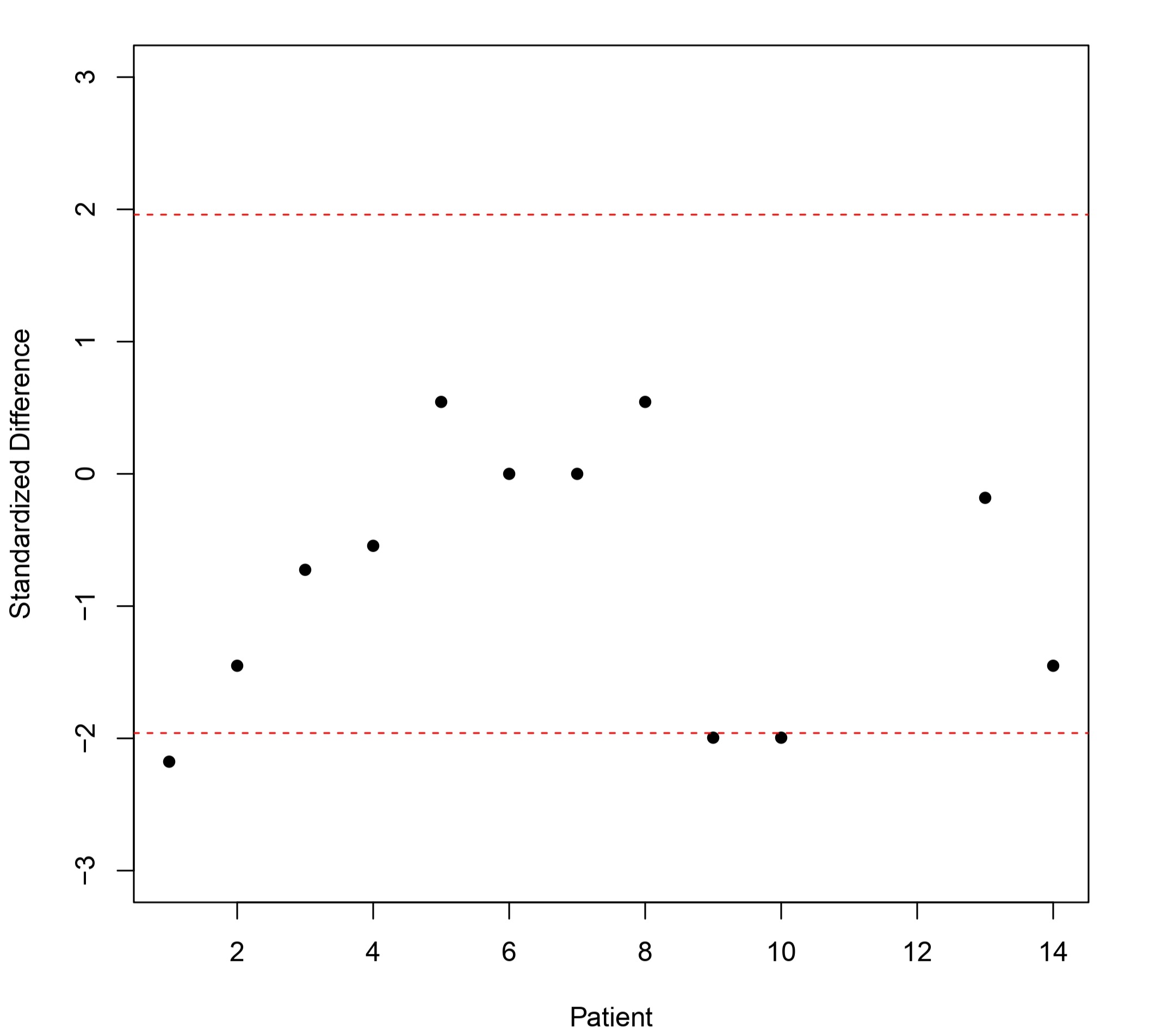


**Note.** Most participants (n = 10) showed Reliable Change Index (RCI) values between −1.96 and +1.96, indicating no reliable clinical change in anxiety symptoms following the intervention. Two participants (Patient 1 and Patient 9) showed RCI values below the −1.96 threshold (−2.176 and −1.995, respectively), suggesting a reliable worsening of anxiety symptoms.

**Supplementary Figure 4.** Reliable and Clinically Significant Change (RCI) Classification for CORE-10 Psychological Distress.
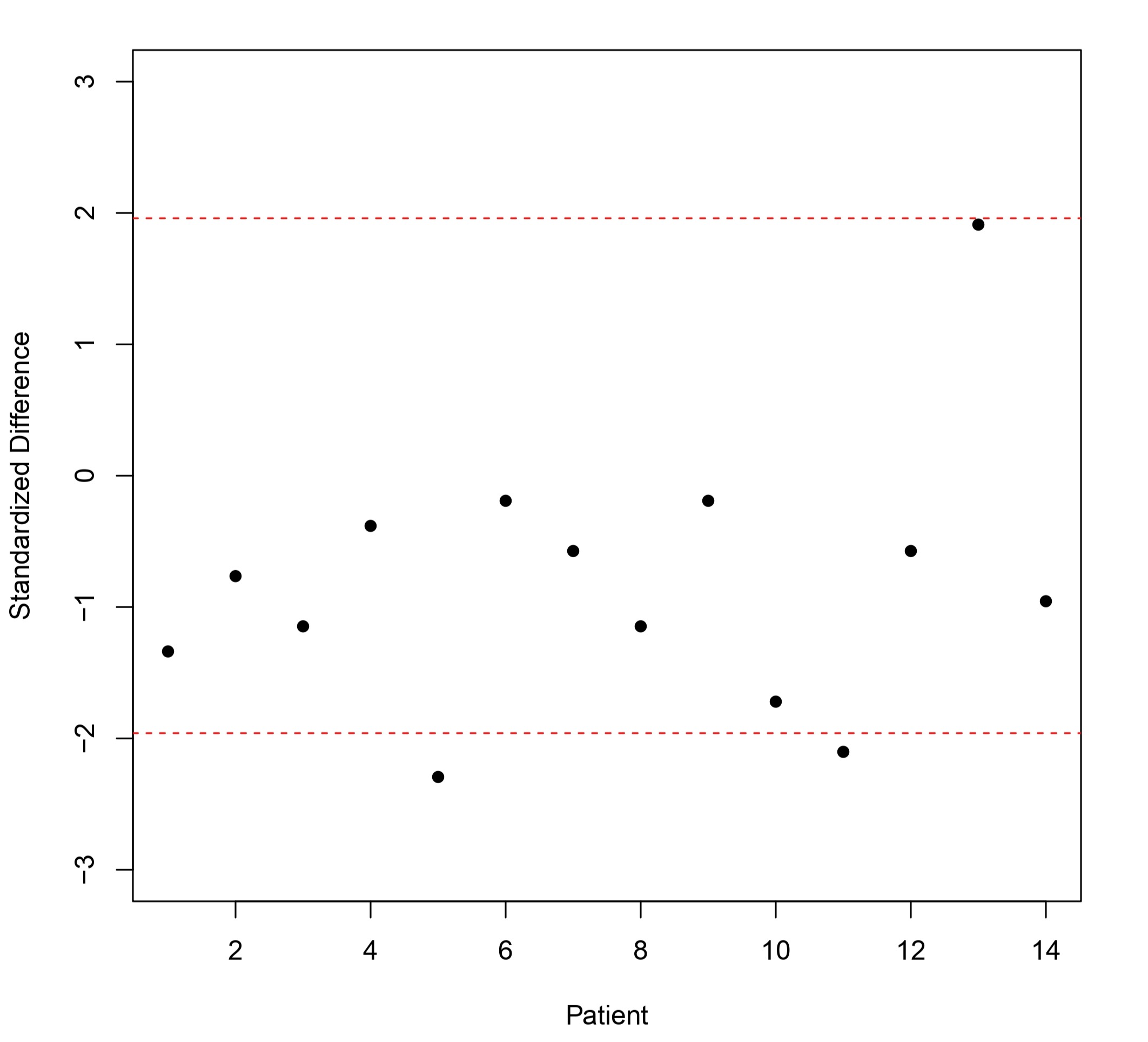


**Note.** Most participants (n = 10) showed Reliable Change Index (RCI) values within the −1.96 to +1.96 range, indicating no reliable change in psychological distress following the intervention. One participant (Patient 13) showed an RCI value close to the improvement threshold (+1.91), suggesting a reduction in distress approaching clinical significance. In contrast, two participants (Patients 5 and 11) exceeded the −1.96 threshold (−2.29 and −2.10, respectively), indicating reliable deterioration in psychological distress.

**Supplementary Figure 5.** Reliable and Clinically Significant Change (RCI) Classification for WHOQOL-BREF Quality of Life.


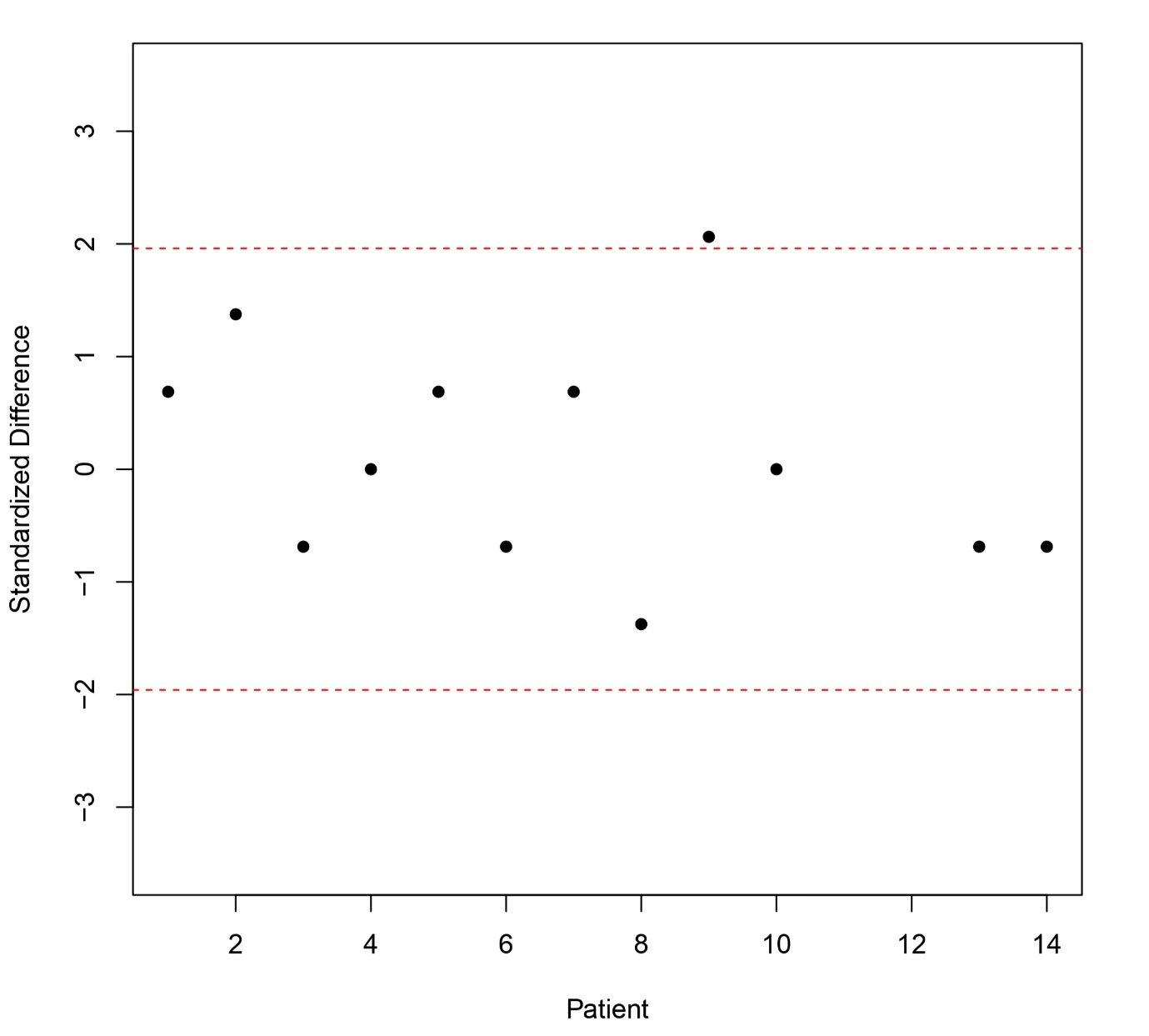


**Note.** Most participants (n = 11) showed Reliable Change Index (RCI) values within the −1.96 to +1.96 range, indicating no reliable change in quality of life following the intervention. Individual scores ranged from −1.38 (Patient 8) to +1.38 (Patient 2), reflecting minor fluctuations that did not reach clinical significance. One participant (Patient 9) exceeded the +1.96 threshold (RCI = 2.06), indicating reliable improvement in quality of life. No participants showed reliable deterioration.**Supplementary Figure 6.** Reliable and Clinically Significant Change (RCI) Classification for MCQ-30 Metacognitive Beliefs.


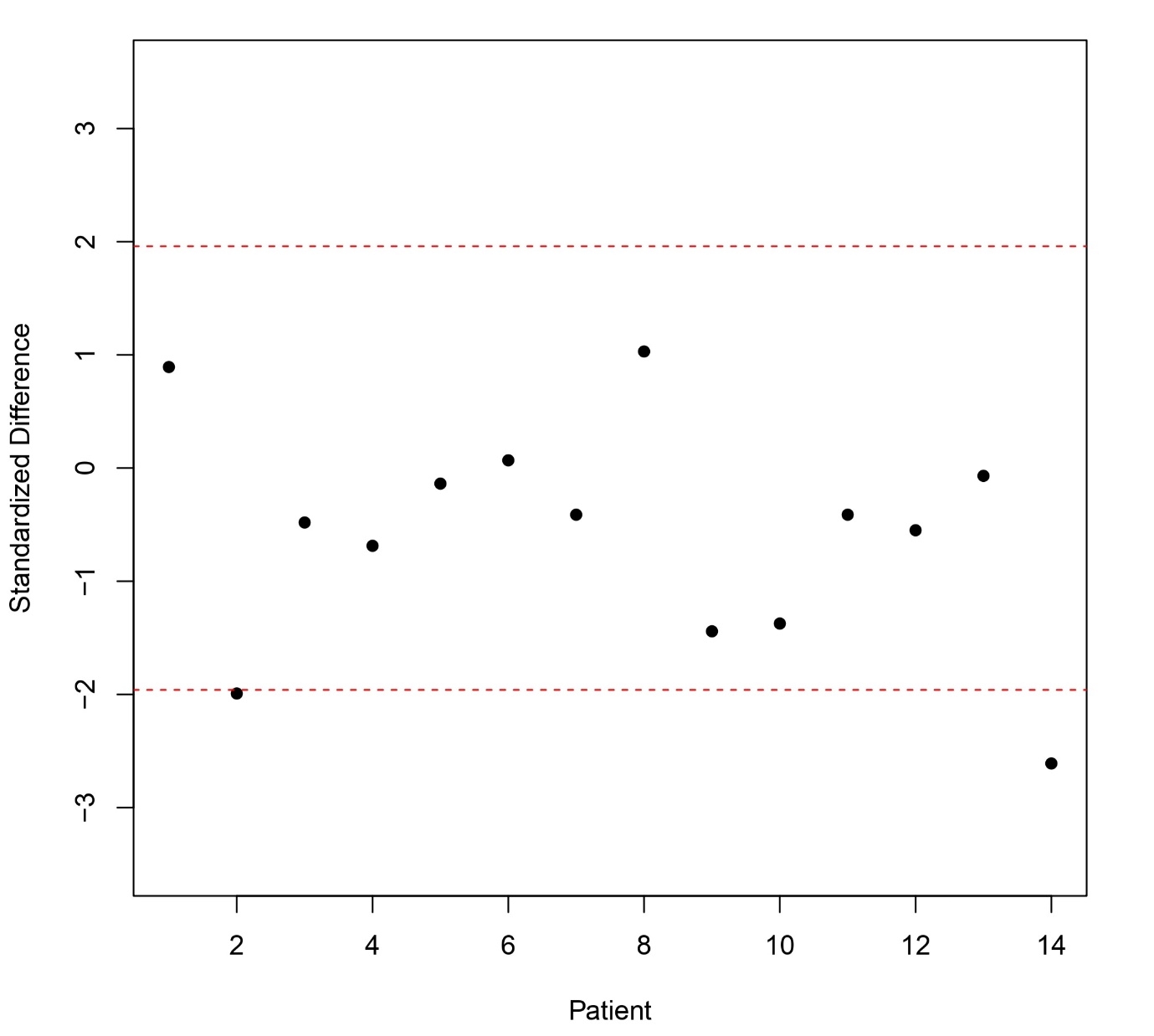


**Note.** Most participants showed Reliable Change Index (RCI) values within the −1.96 to +1.96 range, indicating no reliable change in metacognitive beliefs following the intervention. Individual RCI values ranged from −1.44 (Patient 9) to +1.03 (Patient 8), reflecting minor fluctuations that did not reach clinical significance. One participant (Patient 14) exceeded the −1.96 threshold (RCI = −2.61), indicating reliable deterioration in dysfunctional metacognitive beliefs. No participants showed reliable improvement.

**Supplementary Table 1: Session-by-session MCT+ protocol for individual participants**

| Individualised Metacognitive Therapy (MCT+) | |
| --- | --- |
| **Session 1** | **First Interview** |
|  | Initial meeting to gather patient history. |
| **Session 2** | **Psychoeducation** |
|  | Information and discussion about bipolar disorder, its symptoms, and effects. |
| **Session 3** | **Introduction to Individualised Metacognitive Therapy (MCT+)** |
|  | What is metacognition? Explanation of metacognitive processes.  Identifying individual problems and setting therapeutic goals. |
| **Session 4** | **Case Formulation** |
|  | Individual Case Formulation |
| **Session 5** | **Attributional Style** |
|  | Exploring how a one-sided attribution style can worsen symptoms.  Developing techniques to consider multiple factors in outcomes. |
| **Session 6** | **Decision Making** |
|  | Jumping to Conclusions (JTC): Discussing how premature conclusions can lead to symptoms. |
| **Session 7** | **Changing Beliefs** |
|  | Changing Perspectives 1: Exercises to shift one's viewpoint and beliefs. |
| **Session 8** | **Empathizing** |
|  | Changing Perspectives 2: Learning to understand others' viewpoints.  Implicit Social Laws: Discussing unwritten social rules and applying these insights to daily life. |
| **Session 9** | **Memory and Overconfidence** |
|  | False Memories: Understanding the causes of false memories.  Memory Errors: Identifying different types of memory errors. |
| **Session 10** | **Depression and thinking** |
|  | Identifying symptoms of depression. Exercises to overcome overgeneralization, selective perception, and catastrophic thinking. |
| **Session 11** | **Self-Esteem** |
|  | What is Self-Esteem? Exploring the concept and effects of self-esteem.  Techniques to improve self-esteem and reduce rumination. |
| **Session 12** | **Relapse Prevention** |
|  | Understanding the impact of stigma on relapsing.  Educating others about mental illness and counteracting stigma. |
